# Supplementary figures and images for: Biofilm released cells can easily be obtained in a fed-batch system using ica+ but not with ica- isolates
Source: PeerJ. 2020 Jul 15;8:e9549. doi: 10.7717/peerj.9549 (PMC7368429; doi:10.7717/peerj.9549)

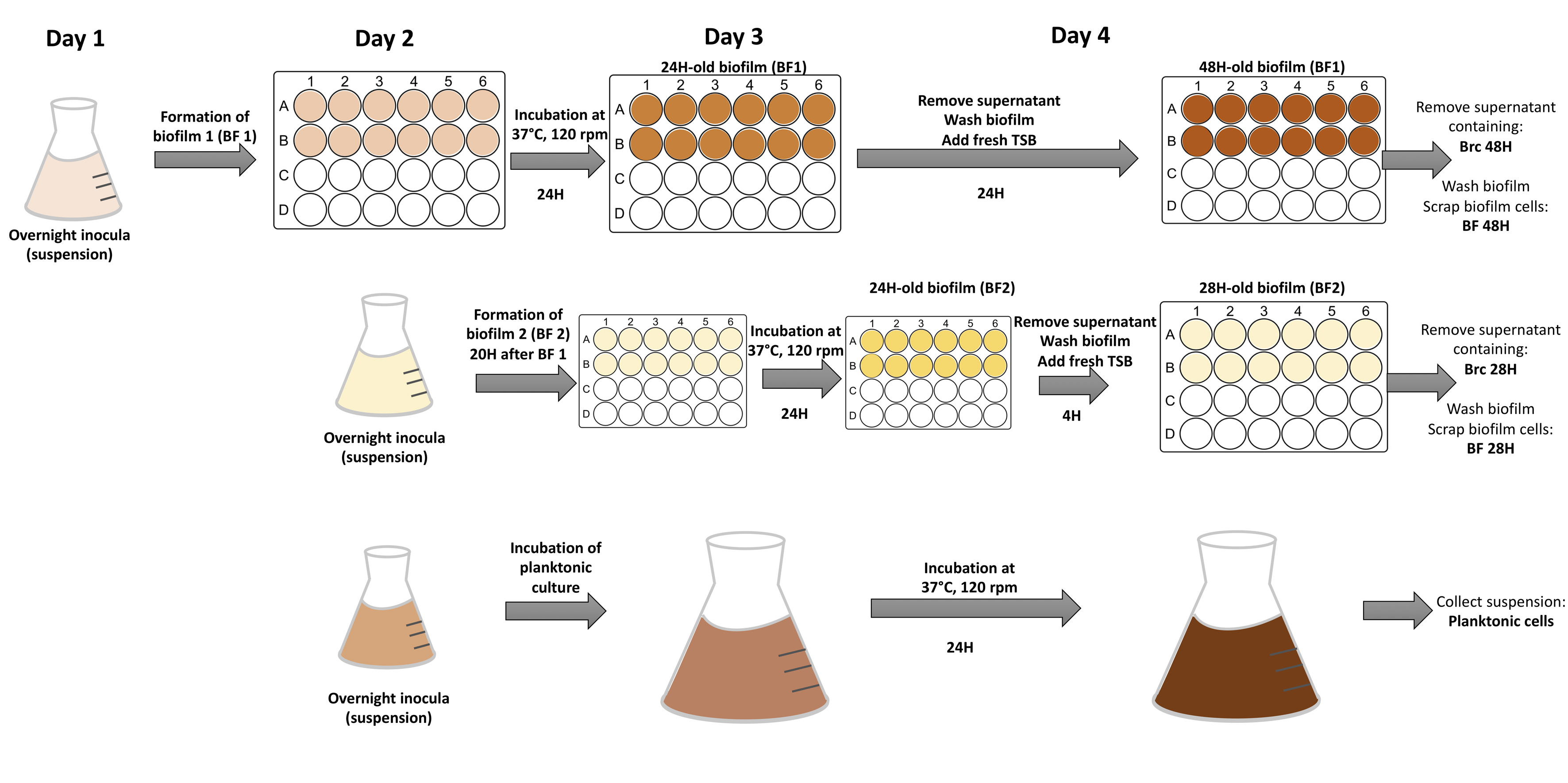

Supplement: Supplemental Information 1 — Bacterial cultures were initiated at different times of the day, in order to obtain all tested conditions at the same time; at the 4th day of each experiment, Brc 28H, Brc 48h, Biofilms and planktonic cultures could be collected simultaneously. [file peerj-08-9549-s001.jpg]

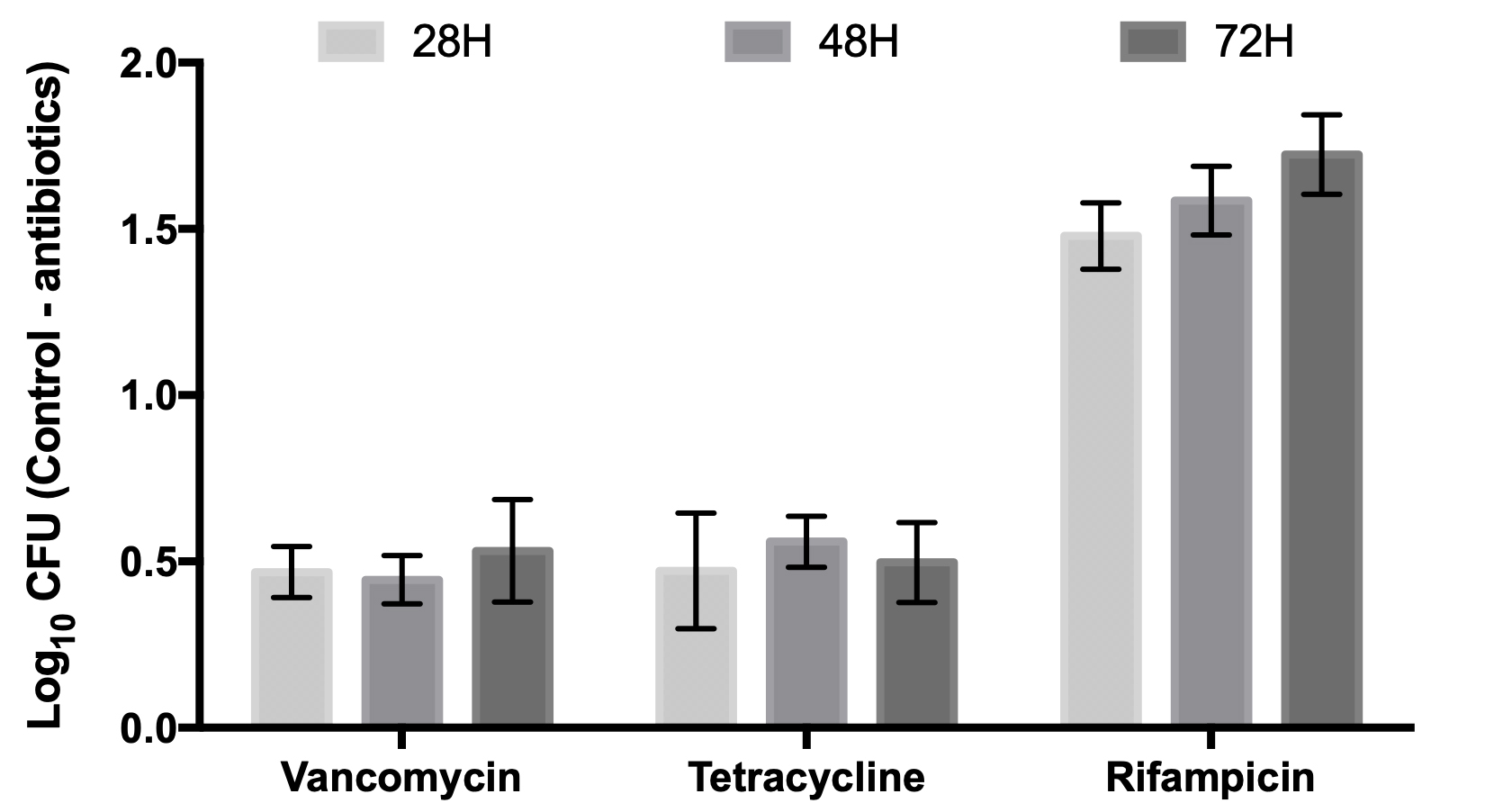

Supplement: Supplemental Information 2 — The columns represent the mean plus or minus standard error deviation of the base 10 logarithmic reduction between samples with antibiotics or just media (controls) of at least three independent experiments. Statistical differences between groups were analyzed with one-way ANOVA multiple comparisons, and no significant differences (p < 0.05) were found among the distinct populations. [file peerj-08-9549-s002.jpg]
